# Metabolic reprogramming landscape orchestrating chlamydospore formation in *Volvariella volvacea*

**DOI:** 10.3389/fmicb.2026.1855276

**Published:** 2026-06-24

**Authors:** Jingjing Ye, Longjin Yang, Jiaqi Qiao, Zihui Pan, Linyan Cen, Fangting Zeng, Mengxin Zhang, Liping Deng, Youjin Deng, Ruoyu Li

**Affiliations:** 1 College of Life Science, Fujian Agriculture and Forestry University, Fuzhou, China; 2 Yunkang Agricultural Technology Company Limited, Sanming, China

**Keywords:** chlamydospore, genome, morphogenesis, transcriptome, *Volvariella volvacea*

## Abstract

Chlamydospore formation in the edible mushroom *Volvariella volvacea* is crucial for its stress tolerance and survival, but its underlying molecular and structural regulatory mechanisms remain largely elusive compared with model fungi. This lack of knowledge hinders efforts to improve stress resistance and advance molecular breeding in this economically important species. To address this, we conducted an integrated analysis combining morphological, ultrastructural, genomic, and transcriptomic approaches across 4 key developmental stages. Our results delineate a clear developmental trajectory of chlamydospore formation: from hyphal swelling to primordium formation, and finally to maturation with a reinforced cell wall. We show that this process is driven by the coordinated activation of storage compound biosynthesis, a shift in energy metabolism, and functional modification of the cell wall. Furthermore, two conserved Velvet family genes, *VvVel1* and *VvLaeA*, act as putative regulators with stage-specific expression patterns. Together, these findings establish a comprehensive metabolic and regulatory framework for chlamydospore development in *V. volvacea* and offer a theoretical basis for stress-resistance breeding.

## 1 Introduction

Chlamydospores are specialized dormant asexual spores produced by fungi in response to environmental stresses such as nutrient deficiency, drought, high temperature, or mechanical damage (Dijksterhuis, 2019). Characterized by thickened cell walls and condensed cytoplasm, they possess extraordinary stress tolerance, serving as a core survival strategy for fungi to withstand adverse environments and perpetuate their populations. Chlamydospores are important structures in fungal taxonomy, genetics, and stress adaptation studies, the regulatory mechanisms underlying chlamydospore development have become a key research direction in fungal developmental biology. Chlamydospores are widely distributed across major fungal lineages, with numerous species in both Ascomycota and Basidiomycota capable of producing these spores, and their biological functions and developmental characteristics exhibit distinct lineage-specific divergence.

With the completion of whole-genome sequencing and the widespread application of omics technologies, significant progress has been made in elucidating the molecular mechanisms of chlamydospore formation, with Ascomycota being the most intensively and systematically studied lineage, encompassing phytopathogenic and biocontrol fungi among other groups. Transcriptomic analyses have confirmed that the induced formation of chlamydospores in ascomycetes is accompanied by dynamic expression changes in a large number of genes that coregulate spore morphogenesis and dormancy traits (Alonso-Monge et al., 2003; Böttcher et al., 2016; Peng et al., 2021; Staib and Morschhäuser, 2005). These genes are involved in cell wall remodeling, lipid and glycogen metabolism, DHN-melanin synthesis, as well as key signaling pathways such as TOR, cAMP, and MAPK. For instance, during chlamydospore formation in the biocontrol fungus *Trichoderma virens*, genes associated with glycogen and lipid metabolic pathways are highly expressed to store energy for spore dormancy, while the upregulation of chitin synthase and glucan synthase genes directly drives cell wall thickening (Peng et al., 2021). In phytopathogenic ascomycetes such as *Fusarium oxysporum* and *Fusarium graminearum*, chlamydospores act as the primary inoculum for soil-borne diseases including banana fusarium wilt and wheat scab, and their formation is also governed by these core metabolic and signaling pathways (Altus et al., 2020; Shin et al., 2024). Additionally, the conserved regulatory complex composed of Velvet family proteins Vel1 and LaeA has been identified as a central global regulator of chlamydospore formation in ascomycetes. This complex integrates environmental signals to coordinate asexual development, secondary metabolism and stress responses (Bayram et al., 2008; Bok and Keller, 2004; Calvo, 2008; Park et al., 2012), Vel1 modulates the temporal rhythm of chlamydospore formation in *Trichoderma virens* (Mukherjee Prasun and Kenerley, 2010), and *LaeA* is even an essential factor for chlamydospore production in *Arthrobotrys flagrans* (Bok and Keller, 2004). Studies on chlamydospores in other ascomycetes such as *Ustilaginoidea virens* and *Candida albicans* have further enriched the molecular understanding of chlamydospore development in this lineage (Yu et al., 2022; Bemena Leo et al., 2021).

In Basidiomycota, chlamydospores are also prevalent in many fungal groups, yet research on basidiomycetous chlamydospores lags far behind that on ascomycetes, with most studies focusing on edible basidiomycetes. This focus arises because chlamydospore formation is closely linked to stress resistance and life-cycle maintenance in edible fungi. Existing studies have confirmed that a variety of economically valuable edible basidiomycetes, including *Sparassis latifolia* and *Morchella* spp., are capable of producing chlamydospores (Li et al., 2020; Yuan et al., 2021). These chlamydospores are formed by cell wall thickening and cytoplasmic condensation of mature hyphae. They possess strong viability under adverse conditions and can germinate to generate new mycelia when the environment becomes favorable. This serves as a critical strategy for these species to withstand harsh environments and maintain population continuity.

*Volvariella volvacea*, also known as the Chinese straw mushroom, is one of the most important edible basidiomycetes widely cultivated in China and Southeast Asia, and is highly prized for its distinctive organoleptic properties and abundant nutritional value (Ali et al., 2024). As a typical edible basidiomycete capable of producing chlamydospores (Miles and Chang, 2004), the chlamydospores of *V. volvacea* exhibit particularly prominent stress resistance, being able to survive for long periods under adverse conditions such as desiccation and extreme cold and germinating to produce new mycelia when the environment becomes favorable (Liu et al., 2020). They serve as a basis for elucidating the yield determinants, senescence mechanisms, and stress resistance regulation of *V. volvacea*. In recent years, the completion of whole-genome sequencing of *V. volvacea* and the application of omics technologies have provided technical support for chlamydospore-related research (Liu et al., 2020; Peng et al., 2021), and preliminary studies have revealed dynamic expression changes of partial genes during chlamydospore formation. However, compared to research on asexual spore development in model fungi, the dynamic metabolic reprogramming network and the key regulatory genes governing chlamydospore ultrastructural development in *V. volvacea* have not been systematically elucidated. Currently, the entire morphogenetic process of *V. volvacea* chlamydospores from hyphal swelling to maturation, especially the fine changes in ultrastructure, lacks systematic elaboration, and the key molecular basis driving this complex developmental program remains largely unknown. Therefore, systematic elucidation of the molecular regulatory network governing chlamydospore formation in *V. volvacea* will facilitate an in-depth understanding of the biological mechanisms underlying stress-resistant structures and dormancy in edible basidiomycetes. It will also provide theoretical insights and genetic resources for molecular breeding. This will enable targeted regulation of chlamydospore formation and promote the advancement of related breeding efforts for *V. volvacea*.

## 2 Materials and methods

### 2.1 Strain culture and sample collection

The *Volvariella volvacea* strain Kangyuan used in this study was obtained by the Edible Fungal Germplasm Resources Management Center of Fujian province, Fuzhou, China. For genome sequencing, the strain was cultured on potato dextrose agar (PDA) plates and incubated at a constant temperature of 32 °C for 5 days; fresh hyphae were then collected and immediately snap-frozen in liquid nitrogen for subsequent genomic DNA extraction. For transcriptome sequencing and all subsequent phenotypic, ultrastructural and biochemical analyses, the strain was cultured in PDA slant test tubes. The developmental time windows for chlamydospore formation (S1: 1.5 d, S2: 2.5 d, S3: 4 d, S4: 7 d) were first determined through preliminary time-course experiments under the same culture conditions. Hyphal and chlamydospore samples were then collected at these four key stages: 1.5 d (S1, vegetative growth phase), 2.5 d (S2, expansion initiation stage), 4 d (S3, primordium formation stage), and 7 d (S4, maturation stage). To ensure stage purity, prior to each collection every slant test tube was examined under a microscope. Using a sterile inoculation needle, only hyphal areas exhibiting highly consistent morphological features of the targeted stage were carefully scraped, and each sample was immediately rechecked under the microscope. All collected samples were snap-frozen in liquid nitrogen immediately after harvesting and stored at −80 °C for further experimental use.

### 2.2 Sample preparation and observation of scanning electron microscopy (SEM)

Chlamydospore samples were fixed in 2.5% glutaraldehyde solution for 12 h, rinsed 3 times with PBS buffer, dehydrated in a graded ethanol series (30, 50, 70, 90%) for 20 min each, then dehydrated in 100% absolute ethanol for 20 min (repeated 3 times). Subsequently, the samples were replaced with a mixed solution of tert-butanol and absolute ethanol at a volume ratio of 1:1 for 20 min, followed by replacement with pure tert-butanol for 20 min. After supercritical drying, the samples were placed under a scanning electron microscope to observe their microstructure (Boyde and Maconnachie, 1981).

### 2.3 Sample preparation and observation of transmission electron microscopy (TEM)

Hyphal samples were scraped from the test tubes, fixed with 1.5% glutaraldehyde at 4 °C for 24 h, and then rinsed 3 times with 0.1 M phosphate buffer (pH 7.4). The samples were then fixed with 1% osmium tetroxide at room temperature for 1.5 h and rinsed three times with distilled water. After dehydration with a graded ethanol series (30, 50, 70, 80, 90, 100%), the samples were replaced with acetone and infiltrated with epoxy resin gradient (resin:acetone volume ratio 1:3 for 2.5 h; 3:1 for 4 h). The samples were embedded in pure epoxy resin and polymerized sequentially at 30 °C, 45 °C, and 70 °C. Ultrathin sections (70 nm) were prepared, stained sequentially with 2% uranyl acetate and lead citrate (double staining), and observed and imaged under a Hitachi HT7700 transmission electron microscope (Graham and Orenstein, 2007).

### 2.4 Staining of glycogen, lipid, and chitin during chlamydospore formation

We used Lugol’s iodine solution to specifically stain glycogen, after 5 min of staining incubation, glycogen was observed under bright field microscopy (Seong et al., 2008). Diluted Nile red staining solution was used for lipid staining, after 15 min of incubation, lipids were observed under a fluorescence microscope with filters of excitation wavelength 543 nm and emission wavelength 598 nm (Son et al., 2012). Diluted Calcofluor White staining solution was used for chitin staining, after 5 min of incubation, chitin was observed under a fluorescence microscope with filters of excitation wavelength 355 nm and emission wavelength 445 nm (Yu et al., 2008).

### 2.5 Determination of melanin in *Volvariella volvacea* chlamydospores

For each stage, we ground 5 mg of sample thoroughly in liquid nitrogen and disrupted it by oscillating with steel beads for 30 min. After removing steel beads, the mixture was centrifuged at 15,000 rpm for 5 min; the supernatant was discarded, and the precipitate was collected as chlamydospore wall extract. To the extract, 1 mL of 4 mol/L NaOH was added, followed by water bath at 80 °C for 120 min. After centrifugation at 10,000 rpm, the supernatant was collected, precipitated with HCl, and re-dissolved in 1 mL of 0.1 mol/L NaOH for extraction. A mixture of 0.1 mol/L NaOH and distilled water served as the blank control. Absorbance was measured colorimetrically at 217 nm (Mao et al., 2010; Wu et al., 2007). All measurements were performed with three biological replicates.

### 2.6 Determination of trehalose in *Volvariella volvacea* chlamydospores

We weighed 10 mg of sample for each stage and determined the trehalose content in *Volvariella volvacea* chlamydospores according to the manufacturer’s instructions of the Trehalose Content (Enzymatic Method) Assay Kit (Shanghai Enzyme-Linked Biotechnology, Shanghai, China). Three biological replicates were analyzed for each stage.

### 2.7 Genomic DNA and total RNA extraction and sequencing

Genomic DNA was extracted from the snap-frozen hyphal samples (for genome sequencing) using the cetyltrimethylammonium bromide (CTAB) method. The sequencing library was constructed with the SMRTbell Express Template Prep Kit 3.0 (Pacific Biosciences, Menlo Park, CA, USA). After library construction, the qualified library was sent to Novogene Technology (Beijing, China) for high-depth sequencing on the PacBio HiFi platform with a sequencing depth of 200 ×.

Total RNA was extracted from the snap-frozen hyphal and chlamydospore samples of the four developmental stages (S1, S2, S3, S4) using Trizol reagent in strict accordance with the manufacturer’s instructions. Three biological replicates were collected per stage. The concentration and integrity of total RNA were comprehensively detected and qualified by agarose gel electrophoresis, Nanodrop2000, Qubit 2.0 Fluorometer, and Agilent 2,100 Bioanalyzer. RNA-seq libraries were constructed using the Truseq™ RNA Sample Prep Kit (Illumina, San Diego, CA, USA). After the constructed libraries passed quality inspection via the Agilent 2,100 Bioanalyzer, the qualified libraries were sent to Majorbio Technology (Shanghai, China) for transcriptome sequencing on the Illumina NovaSeq platform.

### 2.8 Genome assembling and gene prediction

*De novo* assembly of PacBio HiFi reads was performed using the HiFiasm v0.16.1 software (Cheng et al., 2021). Contigs lacking telomeres were manually inspected and joined. The assembled contigs were aligned to the 13 chromosomes of the *V. volvacea* (NF7) reference genome using BLAST software, resulting in the acquisition of 13 chromosomes of the Kangyuan genome. The completeness of the assembled genome was evaluated using the BUSCO software with the basidiomycota_odb10 dataset (Manni et al., 2021). Transcripts were assembled from Illumina sequencing reads using the Trinity v2.6.6 software (Grabherr et al., 2011). With these transcripts as EST evidence and the published protein sequences of *V. volvacea* strain WC 439 as protein homology evidence (Hess et al., 2014), self-training gene prediction was completed using the Maker software (Holt and Yandell, 2011). Meanwhile, gene prediction was performed using the Augustus software with a *V. volvacea* species-specific model self-trained by the Braker software (Brůna et al., 2021; Stanke et al., 2006). Functional annotation of the predicted protein-coding sequences was conducted using the eggNOG-mapper software (Cantalapiedra et al., 2021). Circular layouts were generated with Circos software (Krzywinski et al., 2009).

### 2.9 Repeat sequence and CAZymes identification

Repetitive sequences were analyzed using RepeatModeler and RepeatMasker (Flynn et al., 2020; Tarailo-Graovac and Chen, 2009). Known repetitive sequences were predicted based on the RepBase database, and ab initio repeat prediction was performed using RepeatModeler. The constructed repetitive sequence library was further used for repeat annotation via RepeatMasker.

CAZymes in the *V. volvacea* Kangyuan genome were annotated using the dbCAN3 software (Zhang et al., 2018), in which alignment was performed using the HMMER software with default parameters (E-Value < 1e-15, coverage > 0.35).

### 2.10 Expression quantification and differential analysis

Raw sequencing reads were processed using the fastp software to remove adapter sequences, low-quality sequences, and poly-N containing reads, resulting in clean reads (Chen et al., 2018). Subsequently, the clean reads were aligned to the assembled *V. volvacea* Kangyuan genome using the HISAT2 software (Kim et al., 2019). Based on the alignment results, gene expression levels and fragments per kilobase of transcript per million fragments mapped (FPKM values) were calculated using R packages. Principal Component Analysis (PCA) and sample phylogenetic analysis were performed using R packages to determine the correlation among biological replicates. Differentially expressed genes (DEGs) between samples were identified using the DESeq2 R package. The fold-change was defined as the ratio of expression levels between two samples, and the significance threshold for DEG screening was set as Adjusted *p*-value (padj) < 0.05 and |log2(FC)| > 1.

### 2.11 Gene ontology and KEGG pathway enrichment analysis

Gene Ontology (GO) and Kyoto Encyclopedia of Genes and Genomes (KEGG) pathway enrichment analyses of DEGs were both performed using the ClusterProfiler R package. For both GO enrichment analysis and KEGG pathway enrichment analysis, the significance threshold for enrichment was set as adjusted *p*-value (adjusted by False Discovery Rate, FDR) < 0.05.

### 2.12 Sequence and phylogenetic analysis

Protein sequences of AfLaeA and TvVel1 were obtained from the Uniprot database based on their gene IDs. BLAST was used to align these sequences against the protein sequences of *V. volvacea* Kangyuan. The Conserved Domain Search Service, an online tool from NCBI, was used to search for domains of the candidate sequences, and the positions of the SAM and Velvet domains were identified, respectively. Domain distribution maps were plotted using R packages. Phylogenetic analysis was performed using the MEGA software, and the phylogenetic tree was optimized using the iTOL online website.

### 2.13 Weighted gene co-expression network analysis

WGCNA was performed using the WGCNA R package (Langfelder and Horvath, 2008). Genes with counts > 1 in at least three samples were retained. A soft-thresholding power was selected based on the scale-free topology criterion (R^2^ > 0.9). An unsigned adjacency matrix was constructed and converted into a topological overlap matrix (TOM). Modules were identified by hierarchical clustering using the dynamic tree cut algorithm (minimum module size = 60, merge cut height = 0.25). Module eigengenes were calculated, and Pearson correlations between module eigengenes and developmental stages (S1–S4) were computed. Modules with |correlation| > 0.5 and *p* < 0.05 were considered significantly associated with specific stages.

### 2.14 RT-qPCR validation of transcriptome data

First-strand cDNA was synthesized from 1 μg of Trizol-extracted total RNA. RT-qPCR was performed using PerfectStart Green qPCR SuperMix (TransGen Biotech, Beijing, China). The amplification program was 94 °C for 30 s, followed by 40 cycles of 94 °C for 5 s, 58 °C for 15 s and 72 °C for 10 s. Each sample was analyzed with three biological replicates and at least three technical replicates. Gene expression levels were normalized to the internal control Actin (Kangyuan000248) and relative expression was calculated using the 2 − ΔΔCt method (Livak and Schmittgen, 2001). The expression trends from RT-qPCR were compared with RNA-seq data for validation.

### 2.15 Statistical analysis

All statistical analyses were performed using GraphPad Prism and R software. For trehalose, melanin, and RT-qPCR data, one-way analysis of variance (ANOVA) followed by Tukey’s multiple comparisons test was used to assess differences among the four developmental stages. Data are presented as mean ± standard deviation (SD) from three biological replicates. For transcriptomic data, differentially expressed genes were identified using DESeq2 with a threshold of padj< 0.05 and |log₂(fold change)| > 1. Statistical significance was set at *p* < 0.05 or padj < 0.05 as appropriate.

## 3 Results

### 3.1 Morphogenesis and ultrastructural formation of chlamydospores in *Volvariella volvacea*

**Figure 1:**
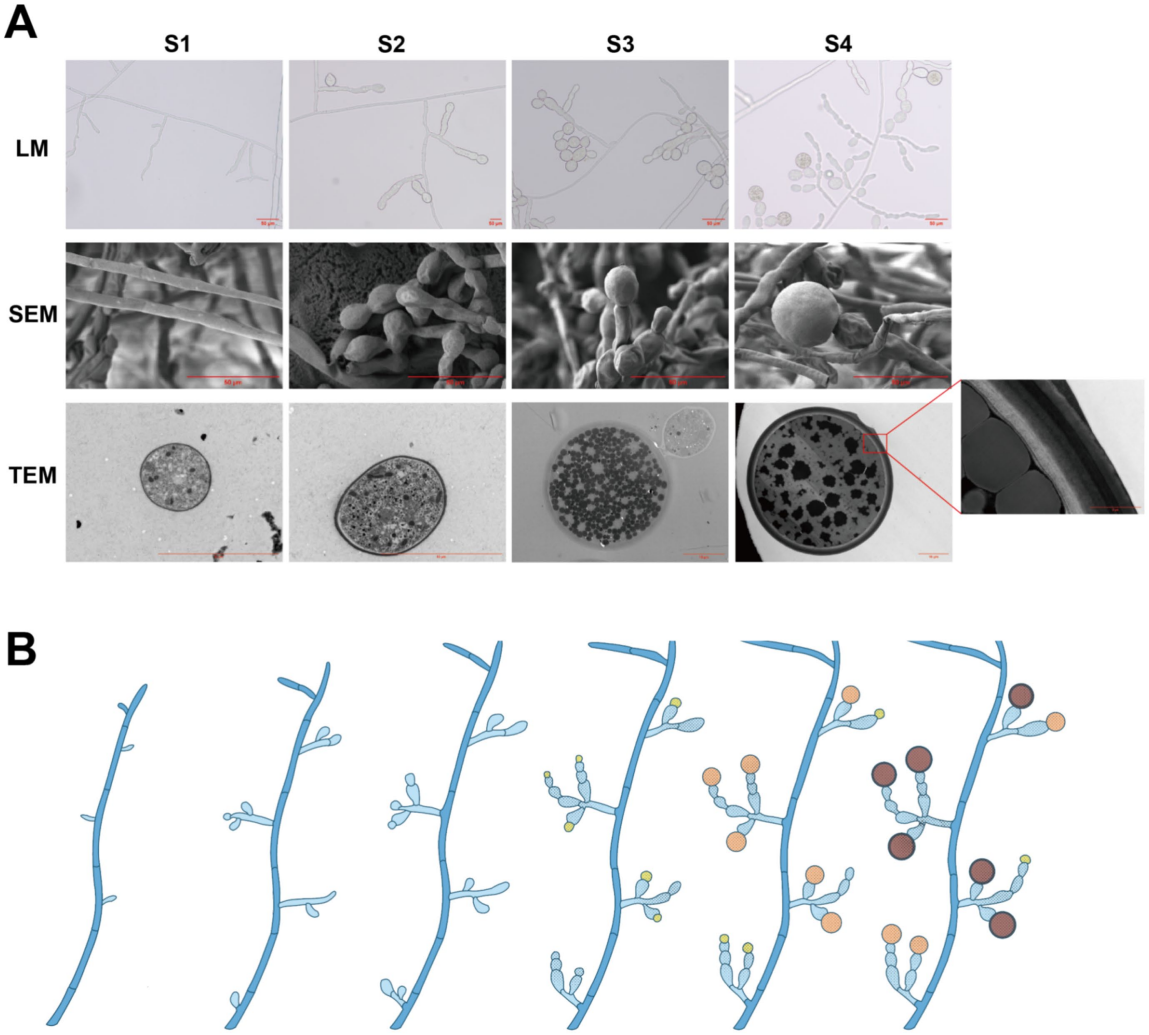
Morphological and ultrastructural characterization of chlamydospore formation in *V. volvacea*. **(A)** Four stages (S1–S4) of chlamydospore formation observed by light microscopy (top, scale bar = 50 μm), scanning electron microscopy (middle, scale bar = 50 μm), and transmission electron microscopy (bottom, scale bar = 10 μm). **(B)** Schematic cartoon model illustrating the progressive developmental stages of chlamydospore formation in *V. volvacea*.

Kangyuan is a wild *V. volvacea* strain, its hyphae can produce abundant chlamydospores when cultured on PDA medium, making it a suitable material for subsequent investigation into the molecular mechanisms underlying *V. volvacea* chlamydospore morphogenesis. We performed a time-course analysis of hyphal development on PDA medium to assess chlamydospore morphogenesis. The initial inoculation stage (S1) corresponds to the vegetative growth phase, characterized by slender, uniform, and smooth hyphae with nascent lateral branches emerging. Subsequently, the culture enters the expansion initiation stage (S2): lateral branches elongate progressively to form multi-level ramifications, with prominent swelling at the apices and internodes to generate short elliptical or spherical structures; the cytoplasm appears sparse and loosely distributed, embedding organelles, and the cell surface exhibits slight roughness. Upon transitioning to the primordium formation stage (S3), swollen cells differentiate sequentially along lateral branches, with cytoplasm gradually becoming dense. The contents of apical cells condense, lipid droplets arrange regularly, and these cells differentiate into round chlamydospore primordia, accompanied by marked cell wall thickening. Finally, the maturation and detachment stage (S4) is attained: spores continuously accumulate and condense intracellular contents; pigment deposition on the cell wall commences, with color gradually turning brown, and the cell wall further thickens into six distinct layers with stage-specific pigment deposition. Mature chlamydospores are loosely attached to parental hyphae only by the outer cell wall and eventually detach, eventually detaching from parental hyphae to form morphologically stable mature chlamydospores (Figure 1A).

### 3.2 Genome assembly and annotation of *Volvariella volvacea* Kangyuan

**Figure 2:**
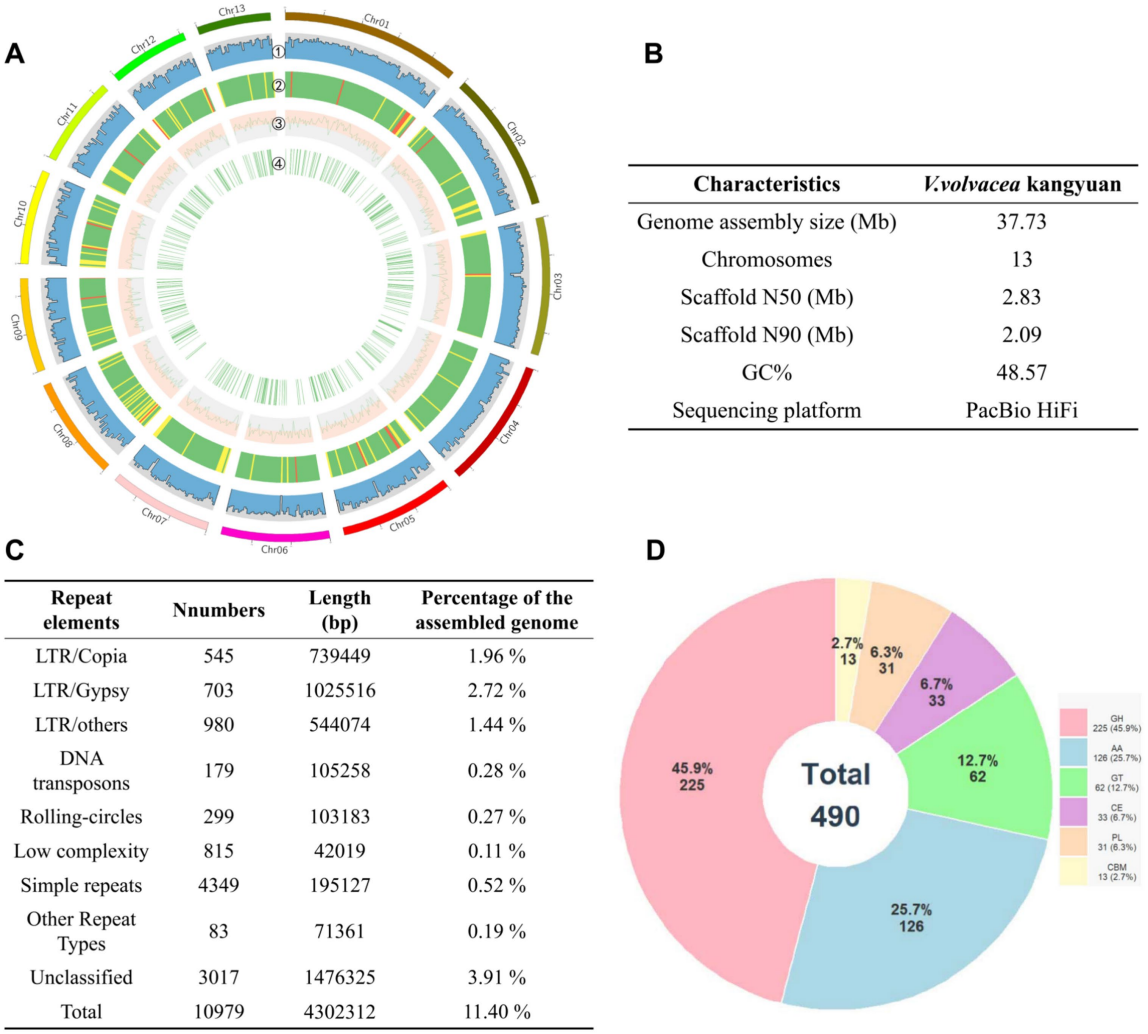
Genomic characteristics and annotation overview of *V. volvacea* kangyuan. **(A)** The outermost layer of colored blocks is a circular representation of the 13 chromosomes, with a scale mark labeling each 1 Mb. ① GC content per window. ② Repeat sequence density per window. ③ Gene density per window. ④ carbohydrate-active enzymes (CAZymes). The window size is 50 kb. **(B)** *De novo* genome assembly and features of *V. volvacea* kangyuan. **(C)** Repeat element analysis in the *V. volvacea* kangyuan genome. **(D)** The distribution of CAZymes categories in *V. volvacea* kangyuan. GH, glycoside hydrolase; GT, glycosyltransferase; PL, polysaccharide lyase; CE, carbohydrate esterase; CBM, carbohydrate-binding module; AA, auxiliary activity.

To investigate the molecular basis of chlamydospore formation, we sequenced the genome of *V. volvacea* Kangyuan using the PacBio HiFi platform. *De novo* assembly yielded a haploid genome of 37.73 Mb with a GC content of 48.57%, assembled into 13 chromosomes (Figures 2A,B), which are consistent with other reported *V. volvacea* genomes. BUSCO assessment showed a completeness rate of 97.8%, and the N50 was 2.83 Mb (Figure 2B), indicating a high-quality genome assembly. A total of 12,313 protein-coding genes were predicted, of which 7,723 were functionally annotated using the EggNOG database. Repetitive sequences accounted for 11.40% of the genome, with LTR retrotransposons being the most abundant (6.12%), followed by DNA transposons (0.28%) and simple repeats (0.52%) (Figure 2C). Furthermore, 490 carbohydrate-active enzymes (CAZymes) were identified, including 230 glycoside hydrolases (GHs), 128 auxiliary activities (AAs), 62 glycosyltransferases (GTs), 34 carbohydrate esterases (CEs), 31 polysaccharide lyases (PLs), and 23 carbohydrate-binding modules (CBMs) (Figure 2D). These CAZymes are likely involved in the dynamic remodeling of the cell wall during chlamydospore formation. Together, these data confirm that we have assembled a high-quality *V. volvacea* genome for exploring the molecular mechanisms underlying chlamydospore morphogenesis.

### 3.3 Synergistic activation of storage compound biosynthesis is associated with reserve accumulation

To explore the dynamic molecular mechanisms underlying *V. volvacea* chlamydospore morphogenesis, we performed transcriptome profiling across 4 chlamydospore developmental stages (S1, S2, S3, S4). Detailed transcriptomic statistics are provided in the Supplementary Results 1.1.

**Figure 3:**
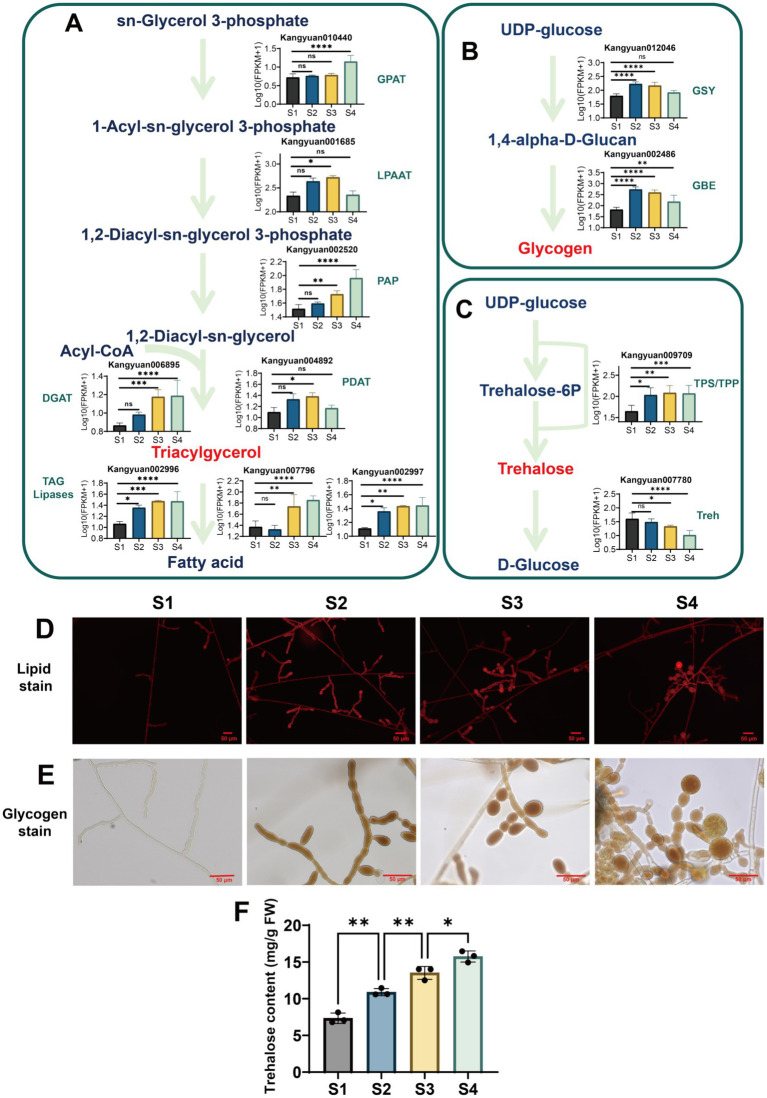
Coordinated activation of triacylglycerol, glycogen and trehalose biosynthetic pathways for reserve accretion in developing chlamydospores. **(A)** Transcriptional profiles of glycerolipid metabolic enzymes and triacylglycerol turnover-related genes. **(B)** Expression dynamics of glycogen biosynthetic enzymes. **(C)** Regulatory gene expression of trehalose synthesis and catabolism. The expression changes of all enzymes in these pathways are represented by bar charts. log10(FPKM+1) values are mean ± SD (*n* = 3). DESeq2 based on raw FPKM values with FDR correction. *padj < 0.05, **padj < 0.01, ***padj < 0.001, ****padj < 0.0001; ns, not significant. **(D)** Dynamically staining lipids with nile red in chlamydospores of *V. volvacea* Kangyuan. **(E)** Dynamically staining Glycogen with Lugol’s iodine solution in chlamydospores of *V. volvacea* Kangyuan. **(F)** Trehalose content (mg/g FW) in chlamydospores of *V. volvacea* Kangyuan. Raw data are provided in Supplementary Table S3, data are mean ± SD (*n* = 3). One-way ANOVA with Tukey’s *post hoc* test: **p < 0.01, *p < 0.05.

Consistent with the cytoplasmic condensation and reserve accumulation (Figure 1), transcriptomic analysis revealed the synergistic activation of storage compound biosynthesis pathways during chlamydospore formation. The triacylglycerol biosynthesis process in the glycerolipid metabolism pathway was upregulated during chlamydospore formation, among which the expression levels of genes encoding glycerol-3-phosphate O-acyltransferase (*GPAT*: Kangyuan010440), 1-acyl-sn-glycerol-3-phosphate acyltransferase (*LAPPT*: Kangyuan001685), phosphatidate phosphatase (*PAP*: Kangyuan002520), diacylglycerol O-acyltransferase 1 (*DGAT*: Kangyuan006895), and phospholipid:diacylglycerol acyltransferase (*PDAT*: Kangyuan004892) were upregulated from S2 to S4, significantly enhancing the synthesis and accumulation of triacylglycerol (Figure 3A). Lipid staining of chlamydospores at different developmental stages using Nile red revealed that lipids were visually detectable inside cells as chlamydospores developed, reserving sufficient energy for dormancy and providing motivation for subsequent germination (Figure 3D).

In addition to the glycerolipid metabolism pathway, two genes in the glycogen biosynthesis pathway, encoding glycogen synthase (*GSY*: Kangyuan012046) and 1,4-alpha-glucan branching enzyme (*GBE*: Kangyuan002486), showed upregulated expressions from S2 to S4 stages of chlamydospore formation, promoting the synthesis of glycogen from UDP-glucose (Figure 3B). Furthermore, glycogen staining of chlamydospore samples using Lugol’s iodine solution showed that internal carbon resources appeared as glycogen in chlamydospores as they matured (Figure 3E). As an alternative to lipids, this represents another core metabolic strategy for spores to establish stress resistance, achieve dormancy, and reserve energy for future revival.

Notably, during chlamydospore formation, the gene encoding trehalose 6-phosphate synthase/trehalose 6-phosphate phosphatase (*TPS*/*TPP*: Kangyuan009709) was upregulated, facilitating the synthesis of trehalose from UDP-glucose precursors. Meanwhile, the expression of alpha,alpha-trehalase was continuously downregulated, slowing down trehalose decomposition (Figure 3C). This expression pattern was in perfect agreement with the significant increase in trehalose content (Figure 3F), which in turn enhanced the structural stability and stress resistance of the dormant spores.

### 3.4 Metabolic reprogramming from biosynthesis to structural reinforcement in cell wall development

**Figure 4:**
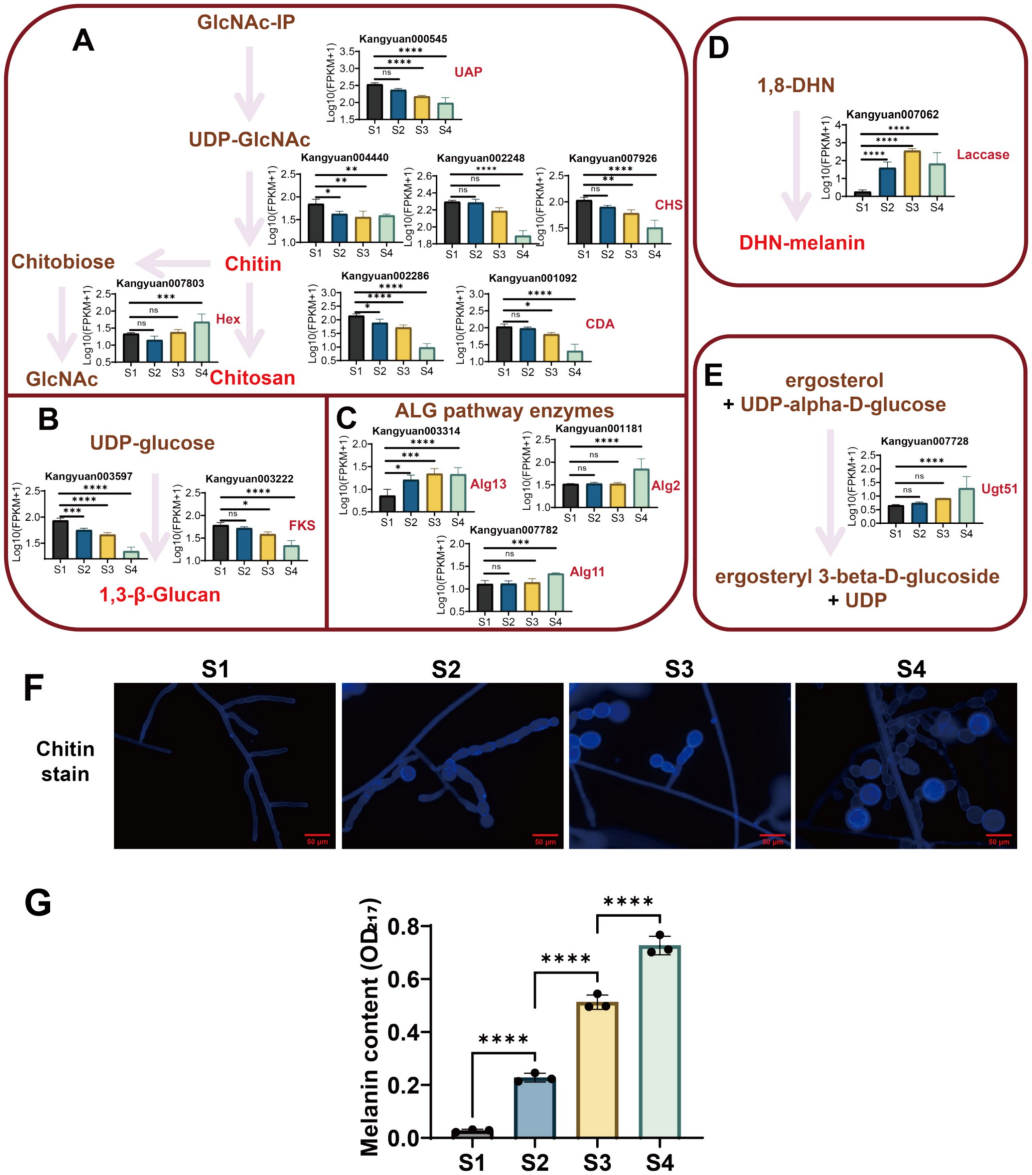
Metabolic shift from cell wall skeleton biosynthesis to functional modification for structural reinforcement in developing chlamydospores. **(A)** Chitin/chitosan biosynthetic and catabolic enzyme expression; **(B)** 1,3-*β*-glucan synthase dynamics. **(C)** N-glycan precursor biosynthesis pathway enzymes upregulation. **(D)** DHN-melanin biosynthetic laccase expression. **(E)** Sterol glucosyltransferase activity. The expression changes of all enzymes in these pathways are represented by bar charts. Log10(FPKM+1) values are mean ± SD (*n* = 3). DESeq2 based on raw FPKM values with FDR correction. *padj < 0.05, **padj < 0.01, ***padj < 0.001, ****padj < 0.0001; ns, not significant. **(F)** Dynamically staining chitin with calcofluor white in chlamydospores of *V. volvacea* Kangyuan. **(G)** Alkaline extraction-based analysis of melanin content (OD₂₁₇) of Chlamydospore in *V. volvacea* Kangyuan. Raw data provided in Supplementary Table S4, data are mean ± SD (*n* = 3). One-way ANOVA with Tukey’s *post hoc* test: ****p < 0.0001.

Microscopic observations during chlamydospore formation showed progressive cell wall thickening, a structural feature that confers stress resistance and stability. Fungal cell walls are primarily composed of chitin, chitosan, and 1,3-*β*-glucan, we thus analyzed the expression dynamics of genes involved in their biosynthetic pathways during chlamydospore formation. Consistent downregulation was observed for the genes encoding UDP-N-acetylglucosamine pyrophosphorylase (*UAP*: Kangyuan000545), chitin synthase (*CHS*: Kangyuan004440, Kangyuan002248, Kangyuan007926), and chitin deacetylase (*CDA*: Kangyuan002286, Kangyuan001092), which attenuated chitin and chitosan biosynthesis (Figure 4A). Interestingly, the expression of β-N-acetylhexosaminidase (*Hex*: Kangyuan007803), a gene responsible for chitin chain trimming, was significantly up-regulated, suggesting a possible remodeling of the cell wall scaffold (Figure 4A). Concurrently, 1,3-β-glucan synthase (*FKS*: Kangyuan003597, Kangyuan003222) was persistently suppressed, reducing 1,3-β-glucan production (Figure 4B). Calcofluor white staining visually detected chitin presence in cell walls across S1–S4 stages (Figure 4F). Notably, hyphal polar growth cessation during chlamydospore formation correlated with downregulated chitin synthesis-related enzymes, consistent with reduced chitin biosynthetic activity.

Despite suppressed chitin biosynthesis, key genes in the N-glycan precursor biosynthesis pathway were significantly upregulated during chlamydospore formation: beta-1,4-N-acetylglucosaminyltransferase (*Alg13*: Kangyuan003314), alpha-1,3/alpha-1,6-mannosyltransferase (*Alg2*: Kangyuan001181), and alpha-1,2-mannosyltransferase (*Alg11*: Kangyuan007782) (Figure 4C). Combined with the up-regulation of *Hex*, these changes suggest that cells actively remodel the existing cell wall scaffold through trimming and modification while promoting extensive deposition of precisely glycosylated wall proteins. This shift enables the construction of a more resilient and structurally refined stress-resistant cell wall, marking a transition from rapid polysaccharide-driven wall assembly to a refinement phase dominated by functional modification and structural reorganization.

During the formation of chlamydospores, DHN-melanin accumulates in the cell wall. Laccase catalyzes the oxidative polymerization of 1,8-dihydroxynaphthalene (1,8-DHN) to form DHN-melanin (Figure 4D). We retrieved the protein sequences of StLac1 and L-Lac1, where StLac1 is a gene that has been demonstrated to regulate DHN-melanin biosynthesis in *Setosphaeria turcica* and L-Lac1 is a laccase with a resolved crystal structure from *Lentinus* sp. Homology alignment against the *V. volvacea* genome identified the candidate protein VvLac1 (Kangyuan007062), which shares 31.56% sequence identity with StLac1 and 57.26% identity with L-Lac1. Structural predictions revealed a high degree of conformational conservation among VvLac1, StLac1 and L-Lac1 (Supplementary Figure S3), which indicates conserved enzymatic function. Notably, *VvLac1* was significantly upregulated during chlamydospore formation, and this upregulation is associated with DHN-melanin deposition in the cell wall and progressive darkening of chlamydospores (Figure 1A). Quantification via alkaline extraction revealed that DHN-melanin content in S4-stage chlamydospores was approximately 26-fold higher than that in S1 (Figure 4G). DHN-melanin enhances chlamydospore stress resistance by modifying cell wall structure and forming cross-links with polysaccharides, thereby reinforcing wall stability.

Additionally, the gene encoding sterol 3-*β*-glucosyltransferase (*Ugt51*: Kangyuan007728) was continuously upregulated during chlamydospore formation (Figure 4E). This enzyme catalyzes ergosterol conjugation with UDP-alpha-D-glucose to form ergosteryl 3-β-D-glucoside, which fills the cell wall “lipid-sugar interface gap”, optimizes cell wall-membrane connections, and enhances overall wall structural stability.

### 3.5 Energy metabolic reprogramming during chlamydospore formation

**Figure 5:**
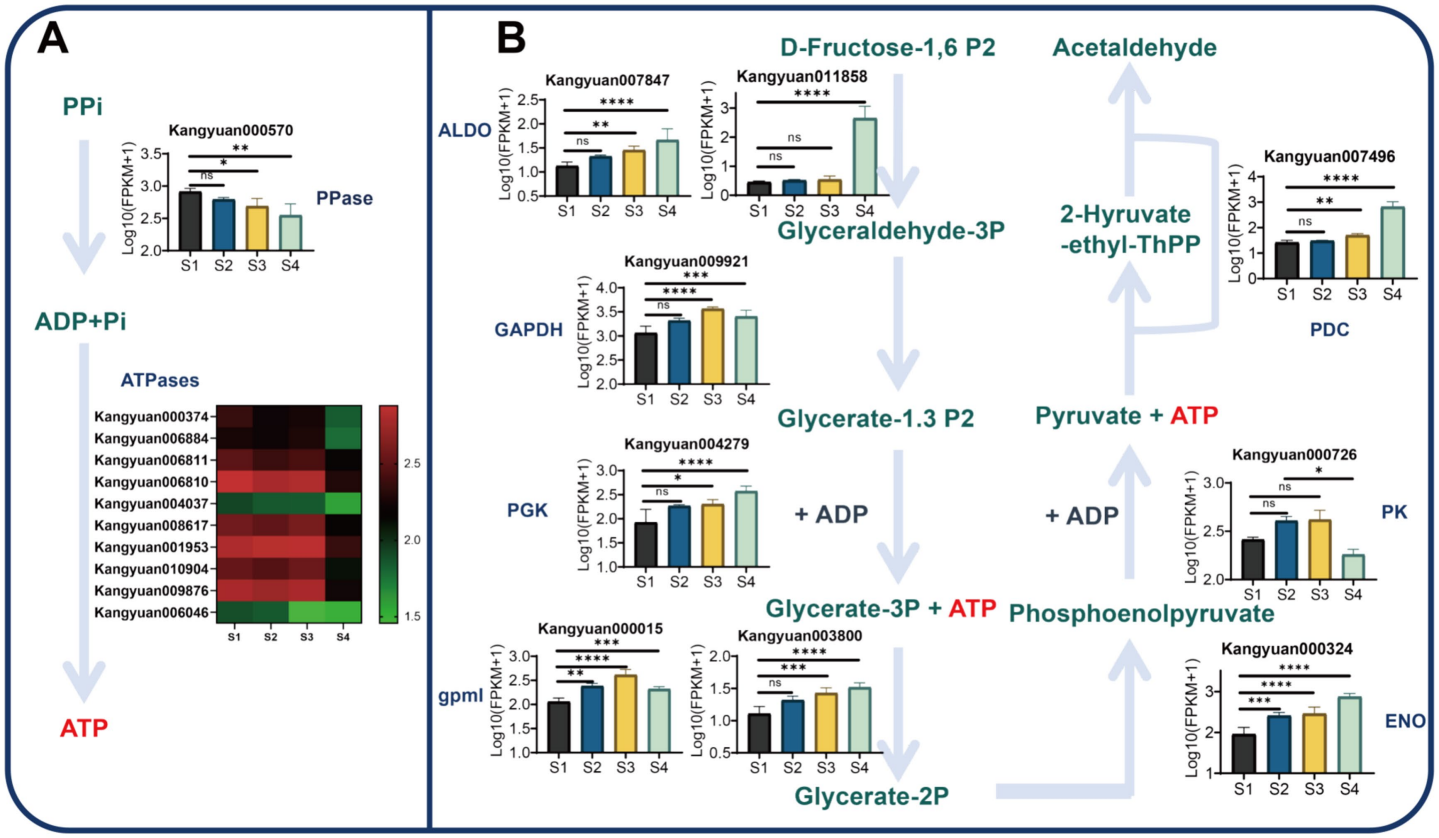
Transcriptional dynamics of energy metabolism machinery during chlamydospore formation. **(A)** Transcriptional profiles of PPase and ATPases linked to oxidative phosphorylation shutdown. **(B)** Stage-specific upregulation of glycolytic core enzymes for chlamydospore morphogenesis. The expression changes of ATPase during the S1–S4 stages are represented by a heatmap, while those of other enzymes are represented by bar charts. Log10(FPKM+1) values are mean ± SD (*n* = 3). DESeq2 based on raw FPKM values with FDR correction. *padj < 0.05, **padj < 0.01, ***padj < 0.001, ****padj < 0.0001; ns, not significant.

Further analysis of all DEGs revealed that the expression of the genes encoding inorganic pyrophosphatase (*PPase*: Kangyuan000570) and ATPases (Kangyuan000374; Kangyuan006884; Kangyuan006811; Kangyuan006810; Kangyuan004037; Kangyuan008617; Kangyuan001953; Kangyuan010904; Kangyuan009876; Kangyuan006046) in the oxidative phosphorylation pathway, a core component of efficient energy production, was continuously downregulated during chlamydospore formation (Figure 5A). This indicates that cells shut down mitochondrial-dominated efficient aerobic respiration and most growth-related anabolic metabolism. However, the core module involving three-carbon compounds in the glycolysis pathway was differentially upregulated during this process: fructose-bisphosphate aldolase (*ALDO*: Kangyuan007847; Kangyuan011858), glyceraldehyde 3-phosphate dehydrogenase (*GAPDH*: Kangyuan009921), phosphoglycerate kinase (*PGK*: Kangyuan004279), 2,3-bisphosphoglycerate phosphoglycerate mutase (*gpmI*: Kangyuan000015, *gpmB*: Kangyuan003800), and enolase (*ENO*: Kangyuan000324) were upregulated to varying degrees of chlamydospore formation (Figure 5B). These genes provide a small amount of energy while generating essential biosynthetic precursors (pyruvate) for chlamydospore morphogenesis and substance accumulation under oxygen-independent conditions. Notably, Kangyuan000726 expression showed a non-significant upward trend from S1 to S3, its marked decrease at S4 coincided with the completion of structural construction in most chlamydospores (Figure 5B), indicating that cells switched from a “construction mode” to a “sequestration and protection mode”. Meanwhile, in the late stage of chlamydospore formation, the upregulated expression of the gene encoding pyruvate decarboxylase (*PDC*: Kangyuan007496) drove the conversion of pyruvate to acetaldehyde, suggesting that the metabolic focus further shifted toward anaerobic adaptation and stress resistance reserve, which provides energy balance and stress protection for chlamydospores dormancy. The reprogramming of energy metabolism may promote a key transition in cellular metabolic mode from active energy production to energy conservation and storage, thereby adapting to the physiological needs of chlamydospores as stress-resistant dormant structures.

### 3.6 Involvement of Vel1 and LaeA as potential key regulators in chlamydospore formation

**Figure 6:**
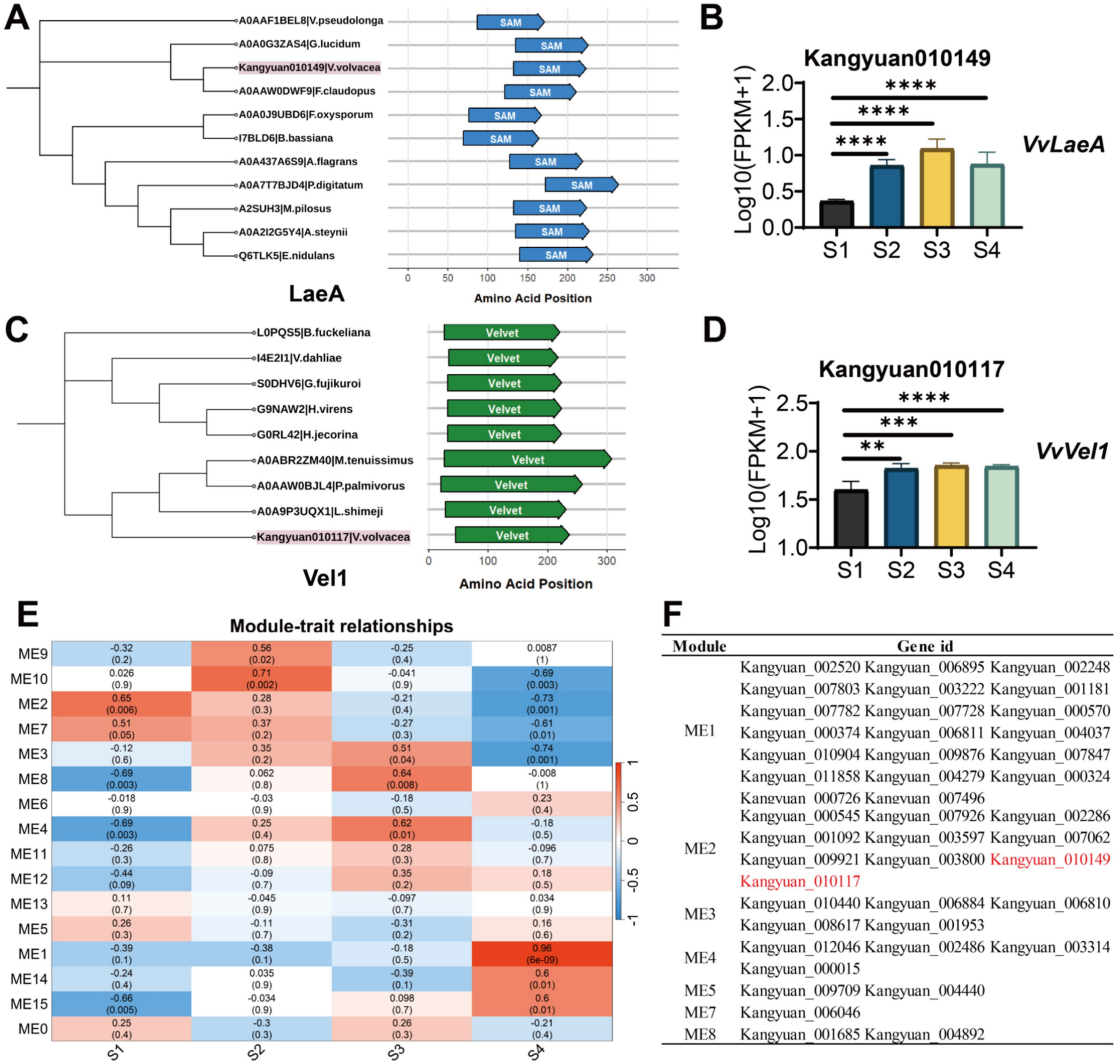
Conserved domains and phylogenetic analysis of Vel1 and LaeA in different fungi. **(A)** Phylogenetic tree of Vel1 proteins and conserved Velvet domain positions in different fungi. **(B)** Dynamic expression of *VvVel1* at different chlamydospore developmental stages. **(C)** Phylogenetic tree of *LaeA* genes and conserved SAM domain positions in different fungi. **(D)** Dynamic expression of *VvLaeA* at different chlamydospore developmental stages. The species and GenBank accession numbers of these Vel1 and LaeA proteins are shown in the figures. Log10(FPKM+1) values are mean ± SD (*n* = 3). DESeq2 based on raw FPKM values with FDR correction. **padj < 0.01, ***padj < 0.001, ****padj < 0.0001. **(E)** WGCNA module-trait correlation heatmap during chlamydospore formation in *V. volvacea*. Values indicate correlation coefficients between module eigengenes and developmental stages, corresponding *p*-values are shown in parentheses. The scale bar on the right indicates the range of possible correlations from positive (red) to negative (blue). **(F)** WGCNA modules of key genes associated with chlamydospore formation in *V. volvacea*.

We propose that the transition from hyphal polar growth to chlamydospore formation is accompanied by decreased synthesis rates of chitin and 1,3-*β*-glucan, the major components of the cell wall, suggesting the activation of a specific regulatory network underlying this morphological transition program. Conserved Velvet factors in filamentous fungi have been widely reported as key positive regulators governing spore formation, maturation, and dormancy. To explore their roles in *V. volvacea* chlamydospore formation, we first identified candidate genes via homologous alignment. Using the global regulator *AfLaeA* from *Arthrobotrys flagrans*, which has been confirmed to regulate chlamydospore formation (Zhang et al., 2023), as the query sequence, we performed a BLAST search against the *V. volvacea* proteome and identified a homologous protein, designated as VvLaeA (Kangyuan010149). This protein shared 32.6% sequence similarity with AfLaeA. Further multiple sequence alignment analysis revealed that VvLaeA, similar to LaeA proteins from other fungi, contains a conserved SAM (S-adenosylmethionine) binding domain, implying it may possess methyltransferase activity (Figure 6A). Transcriptomic data analysis showed that *VvLaeA* was significantly upregulated during chlamydospore formation (Figure 6B), suggesting its potential involvement in the transcriptional reprogramming initiating chlamydospore formation.

Adopting the same strategy, we aligned the sequence of TvVel1, a protein regulating chlamydospore formation in *Trichoderma virens*, and identified a homologous protein VvVel1 (Kangyuan01017) in *V. volvacea*, which shared 39.9% sequence similarity with TvVel1. Domain analysis confirmed that VvVel1 contains a typical Velvet domain (Pfam: PF11754), which is highly conserved among Velvet family proteins and represents a specific amino acid region with DNA-binding capacity (Figure 6C; Ahmed et al., 2014; Park et al., 2012). Multiple sequence alignment also supported that VvVel1 belongs to this protein family. Notably, transcriptomic data similarly showed that *VvVel1* was upregulated during chlamydospore formation (Figure 6D).

To further explore the regulatory potential of *VvVel1* and *VvLaeA*, we performed weighted gene co-expression network analysis (WGCNA) on the transcriptomic dataset. A total of 16 modules were identified (Figure 6E). Notably, VvVel1 and VvLaeA were co-localized in the same module (ME2). This module also contained multiple genes previously identified as being involved in chlamydospore formation, including *UAP*, *CHS*, *CDA*, *FKS*, *VvLac1*, *GAPDH*, and *gpmB* (Figure 6F). The eigengene expression of Module 2 was significantly positively correlated with the S1 stage (vegetative growth phase) (*r* = 0.65, *p* = 0.006). These co-expression relationships further support that *VvVel1* and *VvLaeA* may serve as potential upstream regulators of chlamydospore formation in *V. volvacea*. ME1 contained 20 genes related to cell wall modification and energy metabolism, including *CHS*, *Hex*, *FKS*, *Alg2*, *Alg11*, *Ugt51*, *PPase*, ATPases, *ALDO*, *PGK*, *ENO*, *PK*, and *PDC* (Figure 6F). The eigengene expression of ME1 was extremely significantly positively correlated with the S4 stage (r = 0.96, *p* = 6e-09).

In summary, we identified homologous proteins of the key Velvet family member *VvVel1* and its interacting regulator *VvLaeA* in *V. volvacea*. Both possess the corresponding conserved functional domains, and their expression and co-expression patterns are closely associated with the chlamydospore developmental process. These results suggest that *VvVel1* and *VvLaeA* may act as putative regulators involved in the chlamydospore differentiation program in *V. volvacea*, serving as a reference for subsequent studies on their specific molecular regulatory mechanisms.

### 3.7 Validation of chlamydospore-associated gene expression by RT-qPCR

To validate the RNA-seq-derived gene expression profiles, RT-qPCR was conducted on 15 genes putatively involved in chlamydospore formation. As shown in Supplementary Figure S4, the expression patterns obtained by RT-qPCR were highly consistent with those from RNA-seq. Notably, while the absolute fold changes measured by RT-qPCR and RNA-seq differed due to inherent technical discrepancies between the two platforms, the overall expression trends were fully support our conclusions, thereby demonstrating the reliability and accuracy of the transcriptomic data.

## 4 Discussion

This study characterized chlamydospore formation in *V. volvacea*, including its morphogenesis, metabolic reprogramming, and potential regulatory modules. These findings provide a framework for understanding the developmental mechanisms of stress-resistant dormant structures in edible fungi. In terms of the depth of mechanism elucidation, this study confirmed that *V. volvacea* chlamydospore formation is a complex process involving the coordinated regulation of metabolic reprogramming and structural remodeling. At the energy metabolism level, cells shifted from oxidative phosphorylation to glycolysis and partial anaerobic pathways, driving a transition from an active growth mode to an energy-saving dormant state. This pattern shares commonalities with the energy regulatory strategies of chlamydospores in some other fungi (Abdulghani et al., 2023), suggesting that the formation of dormant spores may follow a conserved evolutionary mechanism of metabolic adaptation across different fungal groups. Enhanced amino acid metabolism was also observed, and as a typical stress-resistant compatible solute (Zaprasis et al., 2015), enhanced proline metabolism may directly improve stress resistance (Carter Zavier et al., 2025), while tryptophan metabolites may provide regulatory signals for chlamydospore formation (Luo et al., 2016). Regarding storage compounds, the coordinated accumulation of triacylglycerols, glycogen, and trehalose represents a multi-level strategy for energy storage and stress resistance (Lin and Heitman, 2005; Martínez-Esparza et al., 2009; Zhu et al., 2022). Previous studies have shown that the synthesis pathways of major fungal cell wall components (chitin, chitosan, and 1,3-*β*-glucan) are mostly upregulated during chlamydospore formation in other fungi (Bemena Leo et al., 2021; Peng et al., 2021; Yang et al., 2014; Yuan et al., 2019). However, our findings suggest that *V. volvacea* may employ a different strategy, as these pathways were not upregulated. This transition mode of “skeleton synthesis inhibition-functional modification enhancement”, including glycoprotein modification, DHN-melanin deposition, and ergosterol glycosylation, indicates that coordinated remodeling of the cell wall and plasma membrane is a key link in constructing stress-resistant structures (Gow et al., 2017; Pereira de Sa and Del Poeta, 2022). This suggests a unique molecular logic underlying the cell wall formation of *V. volvacea* chlamydospores. The spatiotemporal correlation between the upregulated expression of *VvVel1* and *VvLaeA* and the chlamydospore formation process suggests that this module may integrate environmental signals to coordinate downstream metabolic pathways and structural genes. This is consistent with the regulatory functions of this module in other fungi, suggesting evolutionary conservation of fungal developmental regulatory mechanisms (Calvo, 2008; Mukherjee Prasun and Kenerley, 2010; Zhang et al., 2023).

Despite the phased progress achieved in this study, there are still many directions that need to be deepened. First, the elucidation of regulatory mechanisms needs refinement: the interaction mode of *VvVel1* and *VvLaeA* and their downstream target gene networks remain unclear. It is necessary to identify their downstream target genes to elucidate the specific mechanisms regulating chlamydospore formation, metabolic reprogramming, and cell wall remodeling. Meanwhile, the perception of the initial signal for chlamydospore formation and its transmission pathway to this regulatory module still need to be explored to fully reveal the signal regulatory pathway. Second, the molecular basis of the observed multi-layered cell wall morphogenesis needs clarification. The chemical composition of individual layers is still undefined. Integrating solid-state nuclear magnetic resonance (ssNMR) with specific antibody-based high-resolution imaging, as demonstrated in prior studies, can achieve the necessary *in situ* analysis to map both polymer interactions and component localization within this complex structure (Ost et al., 2025). In the future, these technologies could potentially be used to analyze the compositional characteristics of the multi-layered cell wall structure of *V. volvacea* chlamydospores and establish the association between morphology and chemical composition. Third, the functional verification of metabolic mechanisms needs improvement: the functions of key metabolic genes predicted by transcriptomics have not been verified by genetic manipulations. It is essential to construct key gene mutants to systematically verify the functions of genes related to energy metabolism, reserve substance synthesis, and cell wall modification, providing direct genetic evidence for the conclusions of metabolic mechanisms.

Theoretically, the results of this study can enrich the understanding of the evolutionary diversity of fungal dormant spore development, provide a reference for comparative studies on the developmental mechanisms of chlamydospores in fungi of different nutritional types, and promote the development of fungal developmental biology. Practically, the identified key genes can be used as molecular breeding targets to help cultivate new *V. volvacea* varieties with good storage tolerance and strong stress resistance, solving industrial pain points. They also provide clear targets for screening regulators of chlamydospore formation. In the future, it is expected to develop targeted regulators to achieve artificial intervention in chlamydospore formation (Pan et al., 2025; Shalaby and Horwitz, 2015), further optimizing the processes of strain preservation and large-scale production. With the development and application of technologies such as single-cell sequencing and spatial transcriptomics, it is expected to achieve molecular analysis of the chlamydospore formation process at the single-cell level and reveal the functional differentiation of different cell types during development (Kuchina et al., 2021). Screening regulators of chlamydospore production using new targets and analyzing the regulatory mechanisms of chlamydospore regulators through structural biology methods can provide new strategies for artificially regulating chlamydospore formation. Meanwhile, combining the developmental mechanism of chlamydospores with processes such as the growth and development of *V. volvacea* and environmental adaptation to explore the intrinsic association between chlamydospore formation and fruiting body development can provide a new perspective for comprehensively deciphering the life activity rules of *V. volvacea*. In conclusion, this study lays a solid foundation for the research on the developmental mechanism of *V. volvacea* chlamydospores. In the future, through the integrated application of multi-disciplinary technologies, it is expected to achieve greater breakthroughs in theoretical mechanisms and industrial applications, promoting the coordinated development of edible fungi molecular breeding and industrial upgrading.

## Data Availability

The raw sequencing reads of the PacBio HiFi genome sequencing data and Illumina RNA seq data are available in NCBI SRA (https://www.ncbi.nlm.nih.gov/sra), accession number PRJNA1445477. The assembled genome sequences are available in the NGDC (https://ngdc.cncb.ac.cn), accession number PRJCA060841.
