# Supplementary material for: Metabolic reprogramming landscape orchestrating chlamydospore formation in Volvariella volvacea
Source: Front Microbiol. 2026 Jun 24;17:1855276. doi: 10.3389/fmicb.2026.1855276 (PMC13343232; doi:10.3389/fmicb.2026.1855276)
Supplement: Supplementary file 1 [file Supplementary_file_1.docx]

Supplemental material

Metabolic Reprogramming Landscape Orchestrating Chlamydospore Formation in *Volvariella volvacea*

Jingjing Ye ^a, #^, Longjin Yang ^b, #^, Jiaqi Qiao ^a, #^, Zihui Pan ^a^, Linyan Cen ^a^, Fangting Zeng ^a^, Mengxin Zhang ^a^, Liping Deng ^a,^ *, Youjin Deng ^a,^ *, & Ruoyu Li ^a,^ *

^a^ College of Life Science, Fujian Agriculture and Forestry University, Fuzhou, China

*^b^ Yunkang Agricultural Technology Company Limited, Sanming, China*

**Table of contents**

| **1. Supplementary results** |
| --- |
| **1.1.** Dynamic Transcriptomes Map the Developmental Trajectory of Chlamydospore Formation |
| **2. Supplementary tables** |
| **Table S1.** Statistical analysis of sequencing results. |
| **Table S2.** Raw absorbance data and calculated trehalose content |
| **Table S3.** Raw absorbance data of melanin. |
| **Table S4.** Primers for RT-qPCR validation of transcriptomic data |
| **3. Supplementary figures** |
| **Figure S1.** Transcriptome Samples Clustering and differentially expressed genes (DEGs) analysis. |
| **Figure S2.** GO and KEGG enrichment analysis of 820 common DEGs of chlamydospore formation in *V.volvacea* kangyuan. |
| **Figure S3.** Structural alignment of VvLac1, L-Lac1 (PDB:3X1B) and StLac1. |
| **Figure S4.** Expression levels of 15 genes across S1-S4 were detected by RT-qPCR. |

| ***1 Dynamic Transcriptomes Map the Developmental Trajectory of Chlamydospore Formation***  We performed transcriptome sequencing on samples from 4 chlamydospore developmental stages (S1, S2, S3, S4). A total of 254,358,554 raw reads were obtained, and 250,384,684 clean reads were generated after filtering. These reads were aligned to the assembled *V.volvacea* Kangyuan genome, with an average alignment rate of 98.30% per group (Table S1).  PCA and phylogenetic analysis showed good reproducibility of transcriptome samples within the same group and significant differences between samples from different developmental stages(Figure S1A,B). Compared with the S1 stage, the number of differentially expressed genes (DEGs) in other stages was as follows: 1,335 DEGs in S2 vs S1, 2,174 DEGs in S3 vs S1, and 4,247 DEGs in S4 vs S1. All comparison groups contained 4,964 differentially expressed genes (DEGs), among which 820 genes were commonly present in the 3 comparisons (Figure S1C). These common DEGs exhibited a specific expression pattern in the S1 stage that was significantly distinct from other developmental stages(Figure S1D).  GO enrichment analysis of these 820 DEGs revealed that in the Biological Process (BP) category: cell wall organization or biogenesis, carboxylic acid metabolic process, and monocarboxylic acid metabolic process were the most significantly enriched GO terms; in the Cellular Component (CC) category: extracellular region, external side of plasma membrane, and plasma membrane part were the most significantly enriched GO terms; while in the Molecular Function (MF) category, catalytic activity, glucan 1,4-alpha-glucosidase activity, and endo-1,4-beta-xylanase activity were the most significantly enriched GO terms (Figure S2A). DEGs were significantly enriched in pathways such as plasma membrane composition, cell wall biogenesis, and carbohydrate metabolism, suggesting that chlamydospore formation involves tight coupling between cell peripheral structure remodeling and energy supply. Furthermore, KEGG enrichment analysis of the 820 DEGs showed that the most significantly enriched pathways were Arginine and proline metabolism, Microbial metabolism in diverse environments, Tryptophan metabolism, Starch and sucrose metabolism, and Glyoxylate and dicarboxylate metabolism (Figure S2B). KEGG enrichment analysis indicated that the formation of *V.volvacea* chlamydospores may be closely related to enhanced amino acid metabolism, carbon metabolism reorganization, and activation of cross-environmental metabolic adaptation mechanisms. |  |
| --- | --- |

**2. Supplementary tables**

**Table S1.** Statistical analysis of transcriptome sequencing results

| **Sample** | **Raw reads** | **Clean reads** | **Q20 (%)** | **Q30(%)** | **Overall alignment rate (%)** |
| --- | --- | --- | --- | --- | --- |
| S1_1 | 18389974 | 18304962 | 99.23 | 96.20 | 98.19 |
| S1_2 | 20154551 | 20060778 | 99.22 | 96.19 | 98.16 |
| S1_3 | 19205720 | 19100883 | 99.18 | 96.13 | 98.29 |
| S2_1 | 22248558 | 22142005 | 99.20 | 96.11 | 98.53 |
| S2_2 | 21641376 | 21542206 | 99.23 | 96.24 | 98.51 |
| S2_3 | 22583282 | 19748616 | 99.24 | 96.36 | 98.53 |
| S3_1 | 21495411 | 21395500 | 99.22 | 96.22 | 98.43 |
| S3_2 | 18175595 | 18100165 | 99.26 | 96.29 | 98.34 |
| S3_3 | 21543417 | 21430396 | 99.19 | 96.16 | 98.28 |
| S4_1 | 24349217 | 24221487 | 99.21 | 96.26 | 98.15 |
| S4_2 | 20213184 | 20108384 | 99.21 | 96.20 | 98.03 |
| S4_3 | 24358269 | 24229302 | 99.15 | 95.91 | 98.15 |

**Table S2.** Raw absorbance data and calculated trehalose content

| **Sample** | **Standard absorbance (Astd​)** | **Blank absorbance (Ablank​)** | **ΔA** | **Trehalose content (mg/g) FW)** |
| --- | --- | --- | --- | --- |
| S1-1 | 0.656 | 0.303 | 0.353 | 7.028 |
| S1-2 | 0.795 | 0.409 | 0.386 | 7.685 |
| S1-3 | 1.006 | 0.597 | 0.409 | 8.143 |
| S2-1 | 0.691 | 0.160 | 0.531 | 10.598 |
| S2-2 | 0.885 | 0.353 | 0.532 | 10.618 |
| S2-3 | 0.871 | 0.297 | 0.574 | 11.456 |
| S3-1 | 0.848 | 0.121 | 0.727 | 14.509 |
| S3-2 | 0.853 | 0.150 | 0.703 | 14.030 |
| S3-3 | 0.748 | 0.121 | 0.627 | 12.514 |
| S4-1 | 0.880 | 0.087 | 0.793 | 15.827 |
| S4-2 | 0.822 | 0.073 | 0.749 | 14.949 |
| S4-3 | 0.918 | 0.093 | 0.825 | 16.465 |

Glucose standard reference values:

Standard absorbance: 0.518, 0.519, 0.517

Blank absorbance: 0.042, 0.042, 0.042

$$\text{Trehalose content (mg/g fresh weight)}\text{=}\text{0.95×Δ}\text{A}\text{trehalose÷}\text{(}\text{Astd-Ablank}\text{)÷W×D}$$

**Table S3.** Raw absorbance data of melanin

| **Sample** | **OD217** | **Sample** | **OD217** |
| --- | --- | --- | --- |
| S1-1 | 0.022 | S3-1 | 0.544 |
| S1-2 | 0.029 | S3-2 | 0.498 |
| S1-3 | 0.032 | S3-3 | 0.496 |
| S2-1 | 0.223 | S4-1 | 0.767 |
| S2-2 | 0.214 | S4-2 | 0.712 |
| S2-3 | 0.246 | S4-3 | 0.702 |

**Table S4.** Primers for RT-qPCR validation of transcriptomic data

| **Gene ID** | **Forward primer (5′→3′)** | **Reverse primer (5′→3′)** |
| --- | --- | --- |
| Kangyuan007780 | ACGACCCTCCTACCCTTAAT | GTATACCAGCGGACAGTGTAATC |
| Kangyuan000015 | GTCGCTGTTGAGGGTTTAGTT | TCCTTGTCGTAGTTGGCTTTG |
| Kangyuan000324 | CGAGCTTCGAGATGGTGATAAG | CTTTGAGACCGGACTTGATGAG |
| Kangyuan003314 | GACAGGGCTTGGACATTGA | GTACCAGAACCAGCATGACTTA |
| Kangyuan002248 | CTGGCGAGCACAACTATGA | CAGTAGGACCAGGAGGATAGAA |
| Kangyuan004892 | CCTATTGAGCTTGGGAGCTATG | GGAGCTCGACAGTGGTTATTT |
| Kangyuan001181 | TTGAAGGTCGTCCTGCTAATC | GATAGCCACAGAACGGGAATAA |
| Kangyuan002520 | GGCGAATCTGAGGAAGAAGAA | GATCCAGGCATACTGGTCATAC |
| Kangyuan002486 | GTACCTGATGCTCGCCAATAA | TACAGGGACGCAAAGAAGTG |
| Kangyuan010117 | TGTGGTTGCTGGTGCTAAT | GACACCCATACCACCGTTATT |
| Kangyuan010149 | CAGACGGAGACCTACGATATAGA | CCATTCCTCTGCCTCTCAATAG |
| Kangyuan004279 | CAGGCCATTCCTCGCTATT | GCCATACCACCGCAAATAATG |
| Kangyuan007062 | CGCTGGAAGCTCCGAATATAA | CCAGCATTGTCAGTAGCAAATC |
| Kangyuan006895 | GAGCAGCCACAACCTACTATAC | ACTTGGACCTAAACGGCTTC |
| Kangyuan007496 | GGGAAGCACAGGCACTATAA | CAGCTCGTCCTTATCCTTAACA |

**3. Supplementary figures**


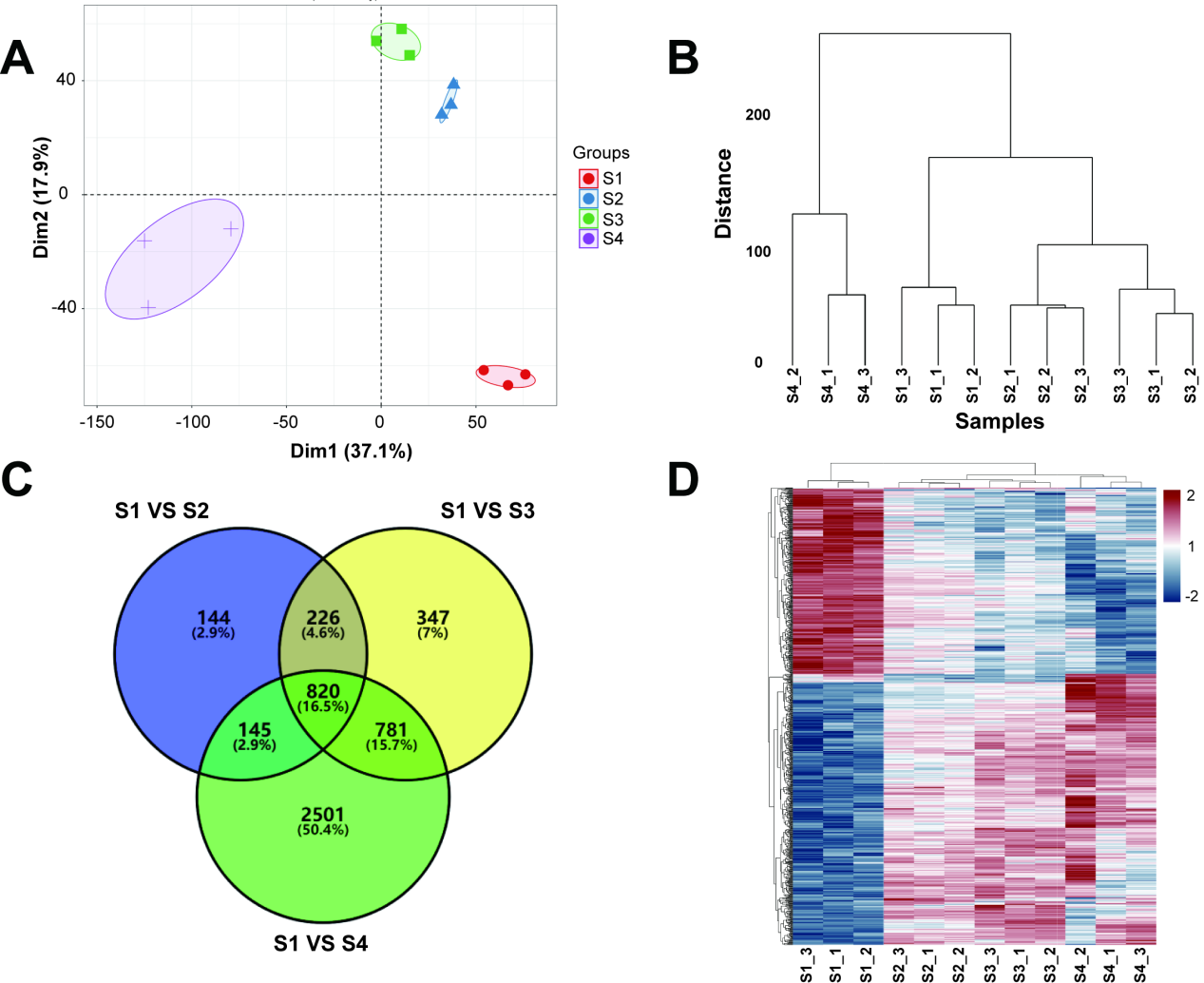


**Figure S1.** Transcriptome Samples Clustering and differentially expressed genes (DEGs) analysis. (A) Principal component analysis (PCA) plot showing clustering of transcriptomes of different *V.volvacea* samples. (B) Phylogenetic analysis of sample correlation coefficients. (C) Venn diagram of unique and common differentially expressed genes (DEGs) between different stages. (D) Clustered heatmap of shared DEGs abundance (log₂(FPKM+0.01)). Red represented a relatively high expression level, and blue corresponded to a relatively low expression level.


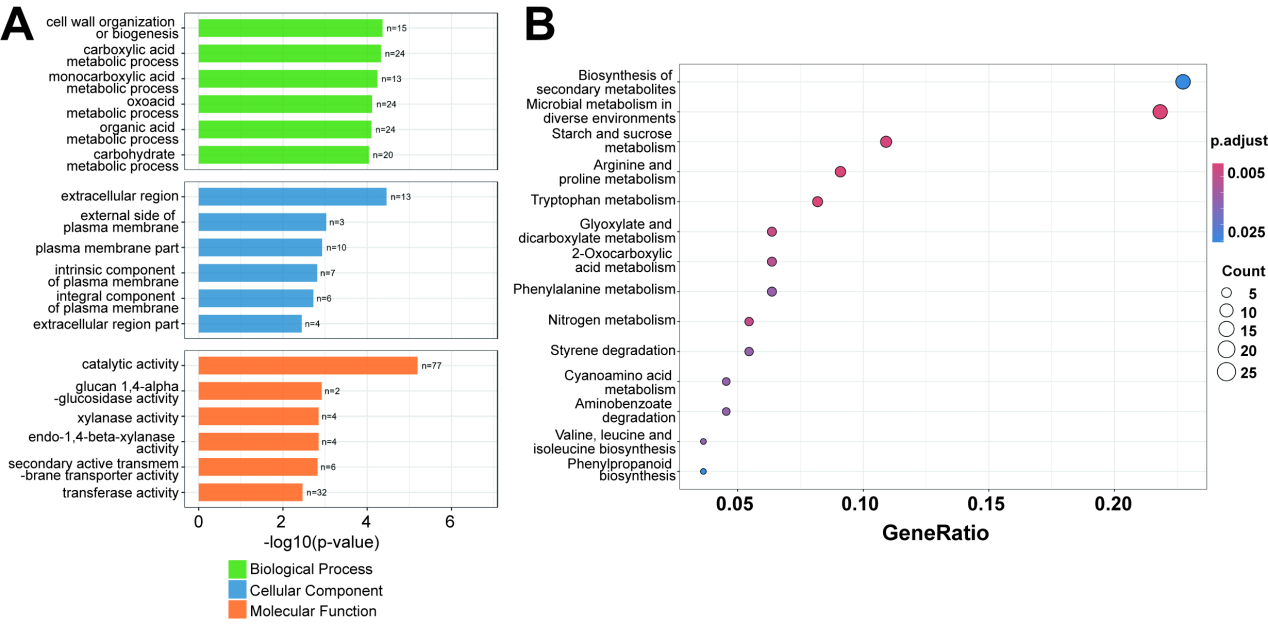


**Figure S2.** GO and KEGG enrichment analysis of 820 common DEGs of chlamydospore formation in *V.volvacea* kangyuan. (A) Top enriched GO terms across BP (green), CC (blue), and MF (orange) categories. (B) Top enriched KEGG pathways.


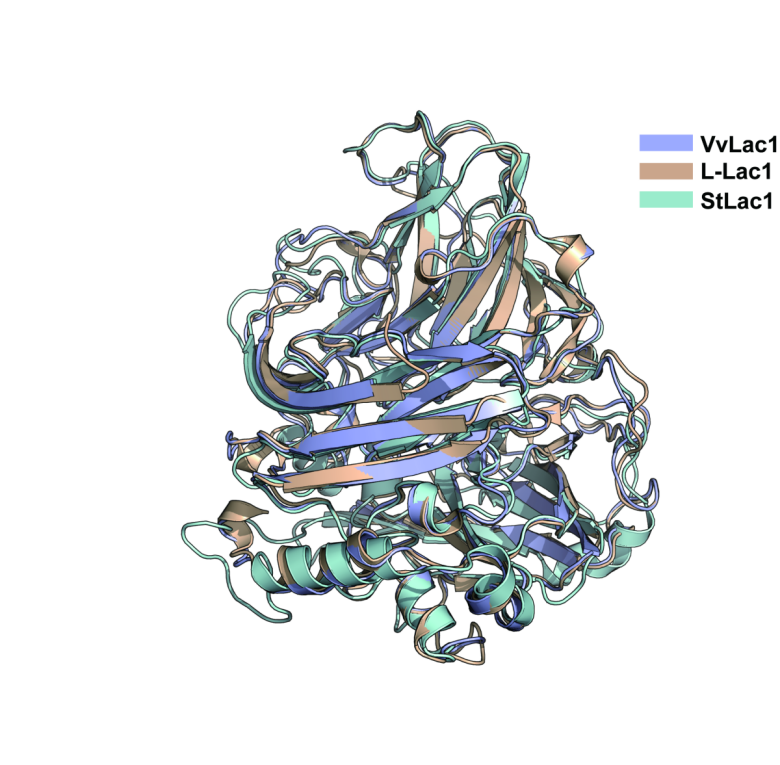


**Figure S3.** Structural alignment of VvLac1, L-Lac1 (PDB:3X1B) and StLac1.


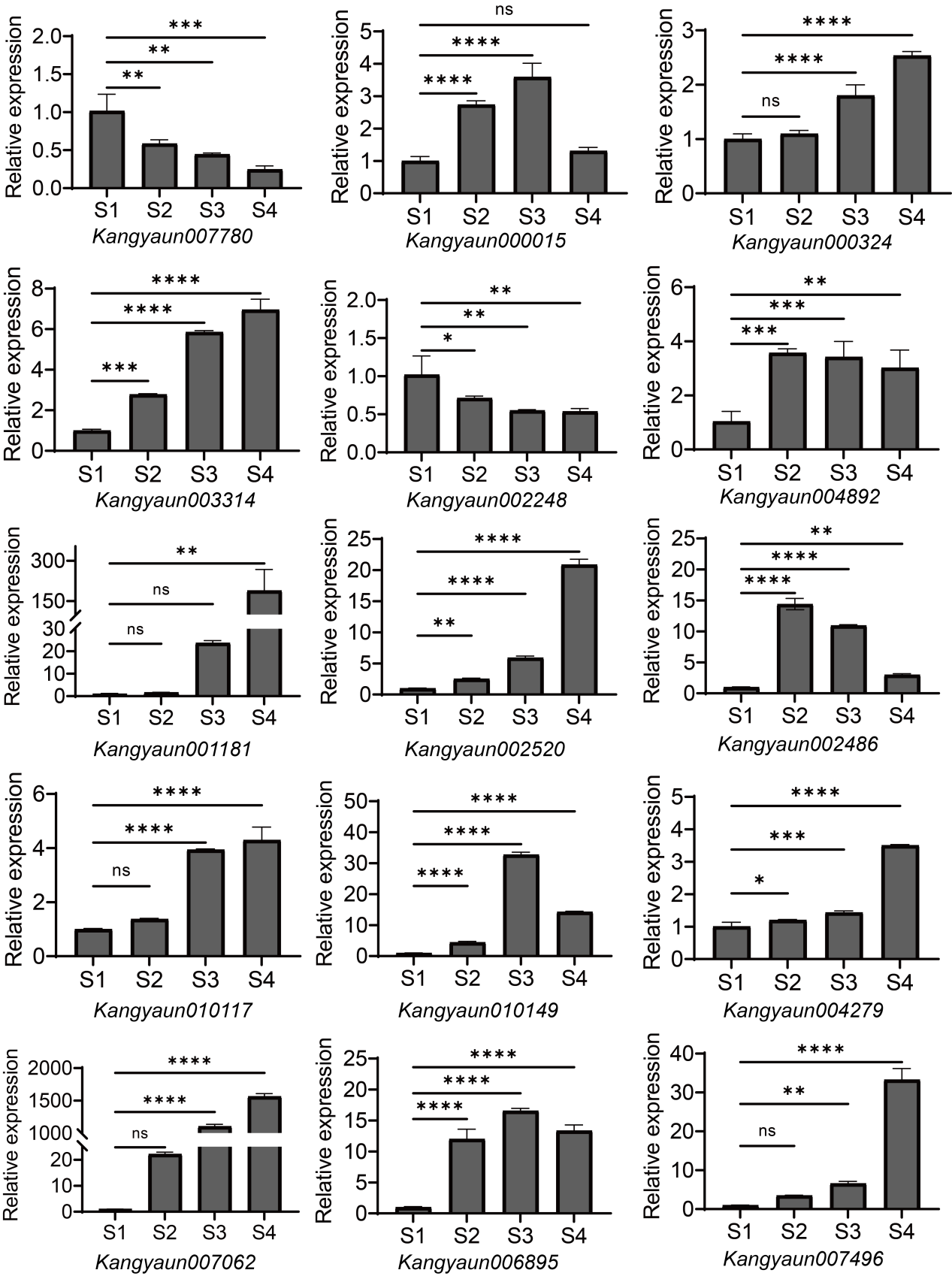


**Figure S4.** Expression levels of 15 genes across S1-S4 were detected by RT-qPCR. Data are presented as mean±standard deviation (SD) of three biological replicates. Statistical differences were determined by one-way ANOVA followed by Tukey’s multiple comparison test. *P < 0.05, **P < 0.01, ***P < 0.001, ****P < 0.0001; ns, not significant.
